# Prospective Evaluation of Fidelity, Impact and Sustainability of Participatory Workplace Health Teams in Skilled Nursing Facilities

**DOI:** 10.3390/ijerph16091494

**Published:** 2019-04-27

**Authors:** Rajashree Kotejoshyer, Yuan Zhang, Marian Flum, Jane Fleishman, Laura Punnett

**Affiliations:** 1 Center for the Promotion of Health in the New England Workplace (CPHNEW), University of Massachusetts Lowell, Lowell, MA 01854, USA; Yuan_Zhang@uml.edu (Y.Z.); Marian_Flum@uml.edu (M.F.); Laura_Punnett@uml.edu (L.P.); 2 Center for Human Sexuality Studies, Widener University, Chester, PA 19013, USA; janefleishman@gmail.com

**Keywords:** occupational safety and health, workplace health promotion, integration, participatory workplace program, process fidelity, program impact, sustainability

## Abstract

Organizational features of work often pose obstacles to workforce health, and a participatory change process may address those obstacles. In this research, an intervention program sought to integrate occupational safety and health (OSH) with health promotion (HP) in three skilled nursing facilities. Three facilities with pre-existing HP programs served as control sites. The intervention was evaluated after 3–4 years through focus groups, interviews, surveys, and researcher observations. We assessed process fidelity in the intervention sites and compared the two groups on the scope of topics covered (integration), program impact, and medium-term sustainability. The intervention met with initial success as workers readily accepted and operationalized the concept of OSH/HP integration in all three intervention facilities. Process fidelity was high at first but diminished over time. At follow-up, team members in two intervention sites reported higher employee engagement and more attention to organizational issues. Two of the three control facilities remained status quo, with little OSH/HP integration. The intervention had limited but positive impact on the work environment and health climate: staff awareness and participation in activities, and organizational factors such as decision-making, respect, communication, and sharing of opinions improved slightly in all intervention sites. Resources available to the teams, management support, and changing corporate priorities affected potential program sustainability.

## 1. Introduction

A number of chronic diseases are known to be associated with psychosocial work stressors, especially low job control, and other organizational factors such as night shift and overtime work [1,2,3,4,5]. It can be argued that because psychosocial job strain is an important predictor of health behaviors, a workplace health promotion (HP) program should seek to improve stressful working conditions in order to support healthy behaviors. Workplace HP programs have traditionally focused instead on trying to modify individual behaviors that increase disease risk. Workplace HP benefits are often unevenly distributed by worker socioeconomic status [6,7,8]. This may be, at least in part, because low-wage, low-status workers face more conditions at work which are obstacles both to the same health behaviors that are HP targets [8,9,10] and to HP participation [11].

A newer approach to workplace HP is that of enhancing its effectiveness by combining it with occupational safety and health (OSH) protections. This concept of integrated employee health programs has been put forward by a few researchers [12,13], the World Health Organization [14], and the U.S. National Institute of Occupational Safety and Health [15]. An integrated strategy has been evaluated empirically by some investigators [16,17,18] but evidence is still sparse as to its effectiveness [19], in part because implementation approaches differ among investigators and thus are hard to compare [20]. Meanwhile, the norm in most workplaces is still that safety programs and workplace HP programs are managed separately.

Healthcare work is physically and psychologically demanding, exposing workers to many workplace stressors that affect their safety and health and which simultaneously may interfere with effective prevention measures [21,22,23]. We designed an intervention for the long-term healthcare sector based on a participatory model that engaged employees in examining and improving the physical, organizational, and psychosocial conditions at work that might impact their health and well-being. The program sought to bridge and integrate occupational safety and health with health promotion by identifying higher-level determinants of employee health and safety [24].

We have previously proposed [9,25] that any workplace health program should involve the workers in a decision-making role, both to ensure that obstacles to workers’ healthy behaviors are recognized and addressed and to increase workers’ decision latitude, a well-known and key health determinant. In a participatory approach, employees are actively engaged in problem identification, program design, implementation and evaluation of the program. The direct involvement of workers in the planning and design of interventions can benefit group and individual self-efficacy, which is consistent with the concept of “sense of coherence” [26], an internal resource for overcoming stress, reducing burnout and other adverse outcomes [27,28]. Participatory ergonomics is one example with demonstrated success as a way to reduce hazardous conditions in the workplace [29,30]. As discussed in depth by Jagosh et al. [31], “partnership synergy” provides a theoretical basis for assessing the links among participatory intervention context, mechanism(s), and outcomes (with elements also commonly utilized in process evaluations).

This article reports our evaluation of process fidelity, extent of OSH/HP integration, health impact, and sustainability of the participatory intervention program. In line with the middle-range “partnership synergy” theory, we have relied on information collected before, during, and after the study to describe the context (institutions and workforce), evidence for posited mechanism of change (e.g., fidelity and amount of intervention), and short- and medium-term outcomes (institutional and individual). The intervention was compared three to four years after initiation in the three participatory intervention program (PIP) sites to three control sites with non-participatory health promotion (NPHP) programs.

## 2. Materials and Methods 

This study, “Promoting Physical and Mental Health of Caregivers through Transdisciplinary Intervention,” was carried out through the Center for the Promotion of Health in the New England Workplace (CPH-NEW), a Total Worker Health® Center for Excellence of the National Institute of Occupational Safety and Health. At the time when this study began, “Total Worker Health” was defined by National Institute of Occupational Safety and Health as “a strategy integrating occupational safety and health protection with health promotion to prevent worker injury and illness and to advance worker health and well-being.” The study was approved by the University of Massachusetts Lowell Institutional Review Board (protocol # 06-1403).

### 2.1. Setting and Sample

The project occurred within the context of a multi-year partnership with a large, for-profit long-term care company, which operated over 200 nursing facilities in 12 states within the eastern United States. In 2003–2006, the company had initiated a “safe resident handling” program in all of its skilled nursing facilities. Some sites also had HP activities as of 2006, depending on local initiative; a corporate-sponsored wellness program began in 2011.

**Figure 1:**
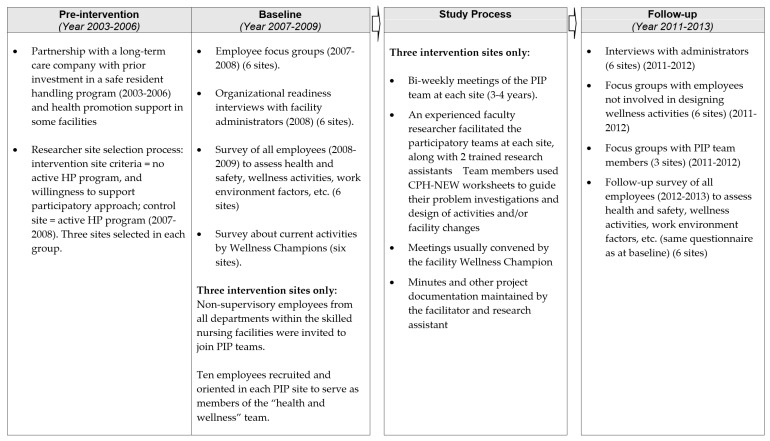
Timeline of the participatory intervention process and impact evaluation. HP, health promotion; PIP, participatory intervention program; CPH-NEW, Center for the Promotion of Health in the New England Workplace.

Within that context, this project component sought to improve the health and safety of nursing home workers by creating participatory teams of non-supervisory workers to address integrated workplace HP and OSH concerns within their own worksites [32]. The teams were started in three skilled nursing facilities in 2008 with the assistance of university researchers. The participatory program was compared with a corporate-initiated wellness program in three other facilities within the same company and geographical region. None of the six facilities had union representation of their workers. We sought to involve all employees, in various job titles and at different organizational levels. Evaluation of program fidelity, impact, and sustainability was based on data from all employees who answered the surveys. Figure 1 provides an outline of the study process and evaluation activities.

### 2.2. Intervention Design

Based on the researchers’ criteria, the company’s regional director for health and safety recommended five skilled nursing facilities that did not yet have active HP programs and whose administrators were expected to be receptive to the participatory intervention process. We selected three of these facilities using a priori criteria to judge which were most organizationally ready for PIP [32]. Another three facilities with pre-existing, corporate-initiated HP programs were recommended by the regional director as control (NPHP) sites on the basis of their current activities and administrator commitment to the program [32]. 

In the PIP centers, team members were recruited from the entire workforce from volunteers responding to posters and management promotion of the program. Each team started with employees from various departments (clinical, dietary, housekeeping, laundry, maintenance, office/business) who met bi-weekly for one hour with two researchers. Initial team meetings involved identification of key issues in workplace health, psychosocial stress, and work organization. 

**Figure 2:**
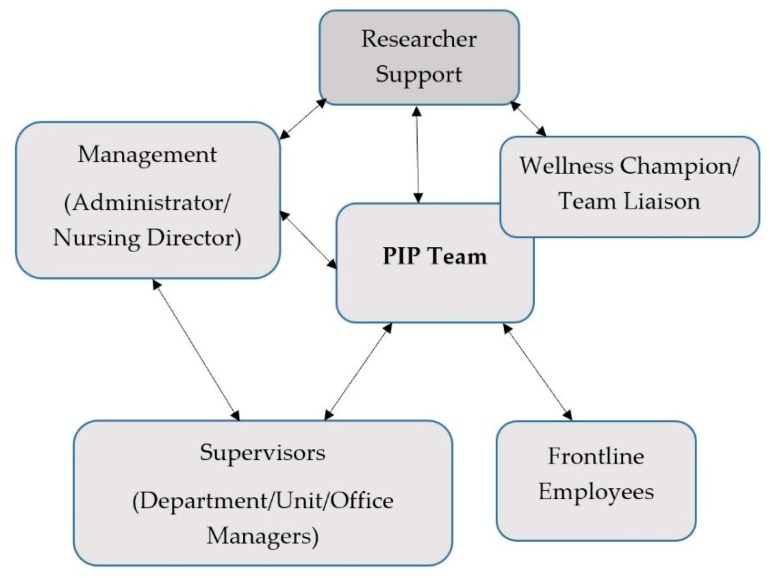
Participatory Intervention Design within Centers.

The intervention began with intensive orientation of the PIP team (2–4 meetings per site over 1–2 months) to worker health and well-being, and Total Worker Health as a comprehensive approach. PIP teams identified issues of importance to members and discussed possible solutions or projects to address these concerns. Team members sought the opinions of their co-workers for program goals and specific activities. PIP team members communicated with individuals at various levels of their facility with updates and available activities (Figure 2). 

Initially the researchers facilitated meetings, guided discussions, and assisted in framing presentations to the site administrator regarding a team’s proposed project. Active facilitation involved the wellness champion and a research assistant attending all bi-weekly team meetings over 2 to 3 years, followed by monthly team meetings over 1 to 2 years, then quarterly telephone check about the program process with the wellness champion per site. The researchers provided technical assistance on a variety of topics, such as seminars on ergonomics in skilled nursing facilities and a food preferences survey to assist in developing programs for healthier food provision. Meeting minutes and activity logs were maintained by the researchers and utilized for ongoing process evaluation. 

The goal was that participatory teams would move over time from co-governance to become independent, with support of the facility wellness champion. Thus, the study plan called for the researchers gradually to reduce our facilitation efforts over time. This was communicated to all participants at the beginning of the project. The phase-out period entailed 1–2 years of quarterly telephone check-ins with the wellness champion.

### 2.3. Data Collection and Analysis

A mixed-method (convergent parallel strategy) approach utilized qualitative and quantitative data to examine the process and impact of the participatory OSH/HP or NPHP program in each facility. Results from the qualitative and quantitative analyses were triangulated with the researchers’ direct experiences and knowledge of the organization to understand the process, impact, and sustainability of the participatory program.

Quantitative data included a brief baseline (pre-intervention) survey of the wellness champions about HP program activities in all six centers. We also conducted employee surveys in the six centers at baseline (2008–2009) and around the fourth year (2012–2013) of the participatory intervention. A self-administered questionnaire collected information on worker chronic disease history, health beliefs and behaviors, and perception of the work environment: physical and psychological job demands, decision latitude, and social support from supervisors and coworkers [8,33,34]. Work environment items included physical exertion, safety climate, psychological demands, decision latitude, and supervisor and coworker support. Psychological demands (two items), decision latitude (two items), physical exertion (five items), and supervisor (two items) and coworker support (two items) were selected from the Job Content Questionnaire (JCQ) [35]. The JCQ subscales have demonstrated good validity and acceptable internal consistency in large study populations from six countries [35]. Safety climate was measured with two items from Griffin and Neal [36] and two items developed by the investigators.

Most analyses compared employees in the pooled intervention (PIP) group to the pooled control (NPHP) group (three centers per group). Baseline (*n* = 645) and post-intervention (*n* = 649) prevalences were compared by cross-tabulation with chi-square statistics and mean values by t-test for independent samples. Cumulative incidence of self-reported chronic diseases was computed from baseline to post-intervention within each group and compared between PIP and NPHP with Fisher exact tests due to small numbers. Within-person changes from baseline to follow-up (limited to workers responding to both surveys) were examined using stratified cross-tabulation and paired sample t-test. All analyses were done with SPSS 22.0 (IBM SPSS, Chicago IL, USA).

Qualitative data included the meeting minutes and activity logs collected throughout the active facilitation period. Follow-up data were collected 3–4 years after the intervention began (2011–2012). Data types included: (1) focus groups with team members; (2) focus groups with other nursing home employees; (3) in-depth interviews with individual team members and wellness champions; (4) in-depth interviews with management (administrators and directors of nursing); and (5) in-depth interviews with supervisors (department heads and unit/office managers) [24].

Other evaluation materials included researchers’ field notes on observed indoor spaces (employee lounge, break room, vending machines, and bulletin boards), outdoor spaces (employee picnic areas and gardens), and printed materials (employee newsletters, flyers, and informational literature) devoted to HP, OSH, or related activities or information. Researcher experiences and observations were logged after each field visit and consulted for purposes of this evaluation. Content analysis was performed using NVivo 9.0 software on transcripts from interviews and focus groups. We compared the themes that emerged across and within the six sites, focusing on (1) integration of HP and OSH, (2) comparative effects of the PIP and NPHP programs, and (3) sustainability of PIP as a model.

Metrics for comparison and evaluation included:

- Process: Fidelity, type and number of activities.
- Integration: Extent to which programs and participants understood and adopted the approach of combined attention to HP (weight loss, exercise) and OSH issues (work environment, psychosocial stressors, ergonomics).
- Impact: Evaluated both at the organizational level (characteristics of the work environment and organization); and at the level of individual staff members (program engagement, awareness, opinions, and participation; health outcomes).
- Sustainability: How long the program lasted, indications of future plans or activities.

## 3. Results

### 3.1. Baseline Site Comparability

In the six centers, a total of 47 interviews were conducted with management (administrators and directors of nursing), supervisors (department heads and unit/office managers), wellness champions, and individual PIP team members. In addition, qualitative data were obtained from three focus groups of PIP team members, three focus groups of employees engaged in wellness at NPHP sites, and eight focus groups with other employees at the six facilities conducted in 2011 and 2012.

According to the baseline survey of wellness champions, and consistent with the criteria for their selection, the NPHP centers had well-developed programs at that time, whereas the PIP centers had emerging programs, i.e., 1–2 activities loosely organized by staff. 

Questionnaires were collected from a total of 645 workers at baseline and 649 workers at follow-up. The PIP and NPHP sites each had more than half of employees in clinical jobs and a predominantly female staff (Table 1). The PIP staff were slightly younger on average. Fewer than 8% in either group indicated fair or poor self-rated health. The average scores were similar for health self-efficacy, prevalence of diabetes and low back problems. Workers in the NPHP centers had slightly higher baseline prevalence of hypertension and elevated cholesterol (Table 1). Decision latitude was higher among PIP staff than NPHP staff at baseline (*p* < 0.01) (Table 2).

### 3.2. Fidelity and Amount of Intervention

The participatory teams were active with the guidance of the researchers. In the intervention sites (I-1, I-2, I-3), the number of regularly attending PIP team members ranged from 4 to 8 of the 10 original members at each site. No NPHP control site (C-1, C-2, C3) had an active team or wellness committee engaging front-line workers.

At the start of this project, not all facilities had a wellness champion appointed. However, by three years after initiation of the program, all six centers had wellness champions, as required under the corporate-sponsored wellness program. Wellness champions were individuals appointed by site administrators to coordinate HP activities in addition to their regular duties. In the PIP centers, these were office staff in ancillary non-supervisory jobs like human resource payroll benefits, medical records manager, and data coordinator. Wellness champions in NPHP centers were all in supervisory positions (assistant admissions director, maintenance manager, and admission director). Interviews and focus groups showed that many staff in the PIP centers knew the identity of their wellness champions. In two of the three NPHP centers, focus group participants were unaware of their wellness champions. 

The three PIP teams met every two weeks for one hour. They were planned to involve only non-supervisory personnel, and they began as such. However, at two of the centers, (I-1 and I-3), they were later expanded by managerial decision to include supervisory and administrative employees. 

PIP teams were guided to utilize the program planning form and project proposals for approval from center administrators. The use of these forms and proposals became less consistent towards the end of the 5 years as some supervisors and managers became members of these participatory teams.

### 3.3. Integration of OSH and HP

In each of the PIP centers, the staff members who joined the teams readily voiced acceptance of the integration concept, i.e., that the obstacles to good health resided both in and outside the work environment. Issues related to both OSH and HP were identified in team meetings. These issues largely mirrored the results of the focus groups at the same centers. The discussions within each team, facilitated by the researchers, demonstrated their operational grasp of the connection between work organization, psychosocial stressors, and personal well-being. Many activities that they carried out represented on-going and systemic attempts to address these concerns in the work environment (Table 3). These included improvements in the workplace through mechanisms for enhanced communication, provision or improvement of employee break rooms and relaxation areas, ergonomics training, and provision of healthy food for staff at a reasonable cost.

At intervention center I-3, when the PIP team spearheaded a gardening project, the original motivation was healthy eating. The team members then also used the garden project as a prototype for developing good proposals and presenting them to management for support and funding. Once it began, they discovered that the garden had many other benefits, including team-building, communications, exercise, stress relief, and a potential for fresh produce for residents as well as staff. 

In contrast, in the NPHP centers, there were no projects designed to address up-stream work organization, psychosocial stressors, ergonomics, or work environment factors. Activities tended to support individual behavior change, e.g., coping, relaxation, and exercise (such as softball games). Correspondingly, none of the interviewed wellness champions in these centers demonstrated an understanding of how features of the job or workplace might influence health behaviors, or any vision as to what integration of OSH and HP might entail. 

### 3.4. Program Impact

At the organizational level, as discussed above, a markedly larger number of activities was carried out in the PIP centers, compared with the control sites. The PIP teams had a number of positive impacts on their health environment at work. All three, independently, addressed lack of healthy food options as a priority and were able to obtain healthier food choices in vending machines. In one facility, the kitchen agreed to provide soups, salads, and sandwiches at reduced cost to employees. One team initiated the creation of a community garden.

The post-intervention surveys demonstrated slightly more organizational changes in the PIP centers than the NPHP centers (Table 4). These included both better communication and more opportunities to voice opinions and influence decisions. More staff members in the PIP sites (28% versus 16% in NPHP sites) said that they were consulted for program suggestions, and in general they reported slightly more opportunities to participate in decision-making and contribute suggestions. Qualitative data (focus groups and interviews) indicated that staff awareness of and participation in team-sponsored activities were higher in PIP centers. Further, researcher notes confirmed that the participatory teams with non-supervisory staff and administrator involvement generated more wellness activities than supervisor-only teams or those with no administrator involvement.

From follow-up survey data, the PIP sites had slightly more employees participating in company exercise (18%) and nutrition programs (25%) than in the NPHP group. There was also notable participation in team-sponsored gardening (9%) and healthy back training (8%), and utilization of outdoor furniture niches set up by the teams for mental relaxation (14%). 

At the individual level, there was no within-person difference in self-reported health status (chronic disease diagnosis, musculoskeletal pain, stress levels, etc.) from pre-intervention to post-intervention in either group (Table 2). There were few notable changes in individual health conditions, health self-efficacy, or selected work factors, and all differences were modest. There was a 6% cumulative incidence of self-reported diabetes in both the PIP and NPHP groups. The NPHP group had a slightly higher incidence of new hypertension (14%) compared to the PIP group (11%). In contrast, the PIP group had a higher incidence of elevated cholesterol (15%) than the NPHP group (11%) (Table 2).

Self-efficacy for eating a healthy diet, avoiding fatty foods, and exercise worsened slightly in both groups over time. The NPHP group gained and the PIP group lost self-efficacy for losing weight, compared to their baseline ratings. Neither group had a change in self-efficacy for managing stress, avoiding smoking or alcohol, and there were no statistically significant differences (*p* < 0.05) between groups for any of these metrics (data not shown).

Decision latitude had been slightly higher in the PIP (*p* = 0.001) than the NPHP group at baseline, while it increased slightly in the NPHP group relative to the PIP group (Table 2). In fact, at follow-up decision latitude was (surprisingly) slightly higher in NPHP than in PIP. This was the only statistically significant change over time in group mean ratings of working conditions. Psychological job demands were reported slightly higher in the PIP group than the NPHP group in both surveys. Coworker support was slightly higher in the NPHP group at baseline and remained stable over time. In contrast, supervisor support decreased slightly in both the groups over time but was slightly better in the PIP group than the NPHP group at follow-up. Ratings of physical exertion at work dropped between the time periods in both groups, which might have reflected the impact of the company’s safe resident handling program [23,37]. The PIP group improved more and had lower physical exertion than the NPHP post-intervention. Workplace safety climate was similar between the two groups and remained stable over time.

### 3.5. Sustainability

Sustainability was examined in relation to organizational conditions of leadership, staff participation, resources, and communication.

**Leadership** was vital for the sustainability of participatory programs. Administrative and supervisor support for wellness towards PIP teams and employees were indicated in many forms within the qualitative data. It included financial support, enabling staff to take time off for meetings and activities, providing meeting space, and verbal encouragement.

Both management and supervisor support were cited in the qualitative data by many employees and managers to be important for program sustainability. The effects of administrator support (and turnover) were mentioned as important in encouraging or discouraging team meetings and activities. Support from management (administrators and nursing directors) and from supervisors (department heads and unit/office managers) were examined separately.

Overall, the NPHP centers demonstrated higher management support than the PIP centers. Administrators in PIP centers discussed HP as part of their everyday work and considered their wellness champions and teams as part of their organizational structure (except in I-2).

Supervisor support was perceived to be higher in two PIP centers, I-1 and I-3, and low at I-2. At the NPHP center C-2, many employees described their supervisors as being supportive to HP by allowing flexibility in their staff schedules and even covering for them while they participated in activities. Other supervisors within this center were described to be supportive by participating in activities themselves and motivating their staff to participate.

PIP teams were able to provide more activities for the staff members with the presence of leadership support. When leadership support was absent, management in these centers talked about being faced with other pressures, needing to make decisions in favor of other more urgent projects and programs.

Management changes in two PIP centers led to combining the existing PIP teams with other, previously inactive committees (“staff appreciation committee” in site I-1, and “fun committee” in I-3). In both cases, the non-supervisory PIP teams were converted to administrator-directed committees with different agendas and priorities.

**Staff participation** in programs and activities is obviously a key measure of program impact as well as likely sustainability. Perceived lack of staff participation in PIP teams and activities was evaluated from the PIP team member interviews and focus groups.

All PIP team members stated in the focus groups that clinical staff had difficulty in getting time to attend team meetings or activities. Staff participation in team-sponsored activities was poor within center I-2. Researcher experience and logs showed that participation in PIP teams had been quite high at the start of the project, yet involvement of clinical staff diminished due to frustrations with their decision-making process and clinical responsibilities.

Employee participation in all three PIP teams dropped to a low number of non-supervisory clinical staff at the end of year 3 of the intervention. Low participation of clinical staff in activities were attributed to staffing shortages, time constraints, and clinical care responsibilities.

**Resources** mentioned in the interviews and focus groups included financial support from the corporation, in-house/in-kind personal effort, and outside support. Although wellness was a stated goal for both the PIP and NPHP centers, no funds were allocated in any of the facilities’ budgets for employee wellness except in the first year (2008), when the corporation provided $700 per year for each facility. After this line item was dropped, most facilities engaged in regular fund-raising for their programs and many of the key informants expressed frustration at the lack of funds for their programs.

In the PIP centers, several projects and especially the higher cost projects simply could not be implemented without adequate resources. At one PIP site (I-2), much of their past activity had focused on fund-raising, which detracted from effort and time that could otherwise have been spent on health and wellness activities.

Using existing in-house resources was an opportunity for the PIP teams to benefit from the knowledge and skills of individual staff members. At Center I-1, one of the nurses offered yoga classes and another offered massages; at I-3, the dietitian offered many in-house programs and services including a weight loss program, potlucks, and healthy recipes.

Team members in the PIP centers identified researcher involvement and guidance as a key outside resource and a vital link to sustainability of the teams. At center I-2, most members believed that the PIP team would not continue without researcher support. The research team provided material support to all three PIP centers, including ergonomic training sessions to staff members, tools and seedlings for the garden, and an exercise class instructor.

Among NPHP centers, the primary forms of outside support cited were discounts for gym membership, health information, and free health screenings by group health insurance companies. In Center C-2, respondents also cited outside support from community programs along with insurance company services.

**Communication** was mentioned in the qualitative data as factors that are essential for sustaining a participatory health program.

Communication between the PIP teams or wellness champions with management as well as with the employees was uneven in both the PIP and NPHP centers. Employees in the PIP centers reported having good communication except at the I-2 center, where both the employees and managers expressed lack of communication as one of the largest stressors and most critical barriers to successful program implementation. The administrator at center I-2 concurred about the lack of communication between the PIP team and management. There appeared to be a trend towards better communication in centers where there was focused attention on work organization issues. In centers where closed-door management meetings were opened to staff or where the wellness champion utilized several methods of communication (e.g., memos, flyers, announcements during meetings, etc.), it was viewed as a smaller problem or not a problem at all.

### 3.6. Differences Among Non-Intervention Centers

Despite the overall group differences, one NPHP center (C-2) exhibited some characteristics and activities similar to the two positive PIP centers from the qualitative data from this center. Even with no official team at this center, the few employees involved in planning the activities had positive management support and were successful in obtaining high staff participation. The administrator, supervisors, and employees appeared to see wellness as a part of their organizational culture. The employee focus group indicated that most of the employees at this center had very few complaints. Most people agreed that good communication existed between management and employees and between the people planning the wellness activities and the rest of the management and staff. While employees at other NPHP facilities appeared uncertain about the idea of integration, the facility manager at C-2 (with previous knowledge about ergonomics and musculoskeletal training) led the safety committee and demonstrated interest in wellness. This manager shared ideas with the researchers about opportunities for integrating OSH and HP within the center.

## 4. Discussion

This study evaluated a participatory, integrated OSH/HP intervention in three skilled nursing facilities compared to three others with more traditional, non-participatory HP programs. Mixed qualitative and quantitative data collected over a four-year follow-up period demonstrated program feasibility, good process fidelity while the researchers were actively involved, meaningfulness of the integration concept to worker representatives, and moderate program impact on some organizational conditions of work. Sustainability, however, suffered due to lack of resources and inconsistent manager support.

### 4.1. Process Fidelity

Process fidelity was high initially in all three intervention sites; the program was introduced in a uniform manner by the researchers and proceeded as intended for the first two years or so. Members of the PIP teams were highly motivated and responsive to the organizing principles of worker priority-setting and a combined focus on both work and non-work obstacles to health. The center administrators permitted front-line staff to volunteer for the teams and assisted with the logistics of scheduling meetings, although use of paid work time for meetings was inconsistent. Involvement of non-supervisory clinical employees in the planning of workplace HP projects was high in the PIP centers at the start of the research project, although it diminished subsequently.

### 4.2. Integration

Compared to the traditional wellness programs in the control sites, the participatory teams in the three PIP centers were far more likely to develop activities with a broad scope, encompassing elements of both OSH and HP. Over time, the teams in the PIP centers addressed work organization, psychosocial stressors, physical ergonomics, in addition to taking an organizational approach to HP goals such as improving the food environment at work. In contrast, the NPHP centers primarily supported individual behavior change, with minimal attention to psychosocial stressors or the work environment. Consistent with our findings, a previous intervention study reported higher blue-collar worker participation with OSH/HP interventions compared to standard HP interventions [16]. In particular, when management’s efforts to reduce workplace hazards were apparent, the workers were more likely to participate [16]. In our study, administrative changes and logistical challenges appeared to cause worker participation in the teams to dwindle gradually.

### 4.3. Impact 

The number and variety of workplace HP activities initiated during the study period were higher in the PIP centers than the NPHP centers. Two of the three PIP centers indicated improvement in organizational factors. There were no larger corporate influences that would have produced these positive changes specifically in the three PIP centers, so it seems reasonable to consider them at least partially as outcomes of the integrated participatory program.

There was no evidence of major change in chronic illness incidence or the perception of health status or behaviors following this participatory intervention, but the four-year follow-up period was too short for any such difference to be expected.

The study hypothesis was that the PIP teams would have more impact than the NPHP programs. In a participatory approach, employees are actively engaged in problem identification, decision making, implementation, and evaluation of the program [38]. This approach has been argued to benefit intervention effectiveness because employees are well-qualified to identify opportunities and obstacles present in their work environment [25], and also because participating in intervention design and implementation could reduce perceived lack of decision latitude [9,39]. Intervention study with assembly workers has demonstrated improved health and work performance in the participatory group compared to controls [40]. While there is substantial literature on participatory workplace interventions, the literature is more consistent about short-term and process benefits than longer-term ones. It remains challenging to compile the evidence in such a way as to identify patterns that explain differences in impact.

### 4.4. Sustainability

Overall leadership support is widely recognized as crucial factors for a sustainable workforce health program of any type [16,32,41] and were so endorsed in the qualitative interviews in our study. Two PIP centers exhibited positive indicators with the participatory approach including essential factors such as support from the center that favored the continuation of meetings and activities. 

Unfortunately, the initially high level of administrator supports and staff participation in project planning diminished somewhat over time. One of our goals was to incorporate the teams into other active committees with similar interests, in order to increase their long-term sustainability. In the two centers where this occurred, the teams were absorbed into committees without responsibility for employee health.

Administrator changes also negatively impacted management support, financial resources, and time release for program participation—all identified as important for progress of the participatory program [32]. Challenges to long-term PIP sustainability included communication barriers among employees, especially in different units, shifts, and job groups; excessive reliance on individual program champions at both site and regional levels; inconsistent corporate commitment to employee HP; and lack of a reward system for champions’ or administrators’ efforts. These mostly pre-dated the participatory teams, although we had sought to screen centers for favorable conditions.

All six centers lacked financial resources to sustain even basic wellness programs, such as paying for instructors in yoga, meditation, or fitness. It did not appear that employee health (other than safe resident handling, which had received a substantial investment) was perceived to generate a high enough return on investment to be sustained in this company.

### 4.5. Study Strengths and Limitations

A major strength of this study is the detailed information obtained from various data sources and the triangulation of qualitative and quantitative data. Mixed methods research is valuable because it captures the information from various perspectives and can support qualitative and quantitative findings [42]. This process evaluation is rich in detail and provides a comprehensive picture of the program. Further, the long follow-up period permits a reasonable understanding of the dynamics over time.

On the other hand, evaluation of the PIP’s impact was limited by the fact that the sites were not selected at random. The three PIP sites were volunteered by their administrators in response to a recruitment effort by the investigators through a key regional staff member. The three NPHP sites were also selected by the same regional representative, in response to the research team’s specified criteria, and then approached for management agreement to permit data collection. As a consequence, there were some anticipated baseline differences between the two groups, both in prior HP activity and possibly in administrator interest in and initiative toward workforce well-being. A further issue is that the study results are not expected to be generalizable, except to other nursing homes with similar management interest and willingness. Nevertheless, the results do demonstrate the feasibility of conducting a participatory change process in this sector, despite (in the U.S.A., at least) tight staffing and scheduling, coupled with low union representation to protect job security for those who voice their opinions about root causes of health and safety problems.

In theory, the gold standard for an intervention study is the randomized controlled trial. However, the benefits of randomization in reducing confounding are not realized except with a large sample. In this case, the intervention was carried out at the level of the entire facility (PIP). In practice, it was not logistically or economically feasible for us to enroll a large number of facilities for such an intervention. Even with some alternative designs, such as the stepped-wedge [43], there is still concern about potential confounding and often a randomization element, thus the number of intervention units remains important. One alternative is to compare the treatment groups on baseline characteristics that might influence the outcome, which we have done here. In fact, perceived working conditions were quite similar except for decision latitude; since that decreased later rather than increasing in the PIP group, the change in time was unlikely to be an artifact of a difference at baseline.

The other advantage of a randomized controlled trial is that with double-blinding, the possibility of information bias can be greatly reduced. However, blinding of participants and researchers is also infeasible for organizational-level interventions. In turn, randomization and blinding may have disadvantages for organizational-level interventions, such as limited capacity to assess multi-dimensional interventions or evaluate process, quality, or performance, and incompatibility with community trust, choice, and participation often needed for successful program design and evaluation [44,45,46].

Another limitation of this study is the difficulty to show the actual impact of the participatory program due to constant changes in the organization. For example, turnover in leadership in the study sites appeared to affect program success with administrator changes in the PIP sites during the study period. Similarly, employee turnover limited our ability to examine within-person changes over time, through reduced statistical power.

Participatory programs can be challenging to implement and evaluate in a research context because key elements cannot be controlled by the investigators; for example, interventions are selected and designed by workers after the program is already underway, and interventions addressing higher-level organizational obstacles may provoke institutional resistance that might not have been visible or even present at the beginning of the project. These issues were known in advance and cannot be prevented even when they are anticipated. It will always be the case that many organizations and workplaces will refuse voluntary worker health improvement efforts, even when resources are offered to facilitate the program. The criteria for selection of participatory intervention sites have been revised on the basis of this experience, in an attempt to better inform decision-makers in advance about the expected process and its potential benefits and costs.

## 5. Conclusions

The intervention program had some positive impacts on work organization in the intervention centers. The fundamental Total Worker Health concept of integrating OSH and HP was intuitive to many workers; they enthusiastically envisioned and sought to carry out integrated programming, and a number of activities improved the health climate in these workplaces. Active involvement of non-supervisory employees in program design and conduct appeared to benefit the work environment and employee morale and engagement. Actively engaged leadership was no less important: program intensity and success fluctuated noticeably with changes in management. Both managers and employees cited the importance for success of factors such as employee program ownership, empowerment, and skill-building (setting appropriate goals, balancing of costs and benefits when weighing intervention alternatives, etc.).

Unfortunately, our planned reduction in researcher facilitation efforts was followed by an erosion of the previously high level of staff participation in project planning. It was disappointing to observe the decline in company commitment to the participatory employee teams despite their demonstrated feasibility and robust worker engagement, in contrast to the company workplace health promotion program.

Participatory OSH/HP is challenging in the long-term care sector due to highly demanding jobs and tight staffing. Managers and front-line workers have different perceptions of the long-term care environment [21], likely arising naturally from their positions in the occupational hierarchy and their consequent exposures to health and safety hazards. Improved systems of communication between levels and program design are needed that support front-line workers to participate in identifying and resolving problems.

PIP team resources, breadth of worker participation, and management support were important preconditions for potential program sustainability. Future efforts should incorporate more robust organizational structures to enhance these factors for program success. Lessons from this study may guide other long-term care facilities to build a sustainable, integrated, and participatory program.

## Figures and Tables

**Table 1:** Baseline characteristics of skilled nursing facility employees (all jobs): 3 participatory intervention program (PIP) and 3 non-participatory health promotion (NPHP) centers.

Baseline Demographics		PIP (*n* = 360)	NPHP (*n* = 285)		
Gender	Female	78.3% (282)	85.3% (243)		
	Male	16.9% (61)	11.2% (32)		
Average Age		39.8 ± 12.4	41.8 ± 12.2		
Nursing Aides *		35.8% (124)	54.3% (120)		
Licensed practice nurse/Registered nurses		22.2% (77)	21.3% (47)		
Other jobs (non-clinical)		41.0% (142)	24.4% (54)		
**Baseline Health Status**		**Cumulative incidence (%)**	**Cumulative incidence (%)**	***p*-value**	**Difference in rates (NPHP vs. PIP)**
Diabetes at baseline		8%	6%	0.77	2%
Hypertension at baseline		18%	20%	0.85	2%
Cholesterol at baseline		13%	25%	0.59	12%

* Jobtitles had missing values of 12.1%.

**Table 2:** Worker health and working conditions in pre- and post-intervention matched pair surveys: Comparison of PIP and NPHP centers.

Health Status	3 PIP centers (*n* = 102)		3 NPHP centers (*n* = 110)		Statistical Significance	
New cases at follow-up:	Cumulative incidence (%)		Cumulative incidence (%)		Difference in rates: NPHP–PIP	*p*-value ^a^
Diabetes	6%		6%		0%	1.00
Hypertension	11%		14%		3%	0.82
High cholesterol	15%		11%		−4%	0.49
Low back problem	8%		8%		0%	1.00
**Work Environment**	**Pre-intervention: mean (SD)**	**Change in mean value (post-pre)**	**Pre-intervention: mean (SD)**	**Change in mean value (post-pre)**	**Mean group difference,** **NPHP – PIP, in post-pre change** **(95% CI) ^b^**	
Health self-efficacy	26.5	−0.87	26.1	−0.30	0.58 (−0.83–1.99)	
Supervisor support	5.69	−0.07	5.87	−0.35	−0.28 (−0.76–0.20)	
Coworker support	5.88	−0.08	5.99	−0.06	0.02 (−0.34–0.37)	
Safety climate score	2.90	−0.30	2.93	−0.32	−0.03 (−0.16–0.11)	
Decision latitude	5.48 *	−0.22	5.18	0.56	0.77 (0.42–1.13) *	
Psychological demands	5.73	−0.11	5.54	0.00	0.11 (−0.22–0.45)	
Physical exertion	11.21	4.90	11.44	5.69	0.79 (−0.22–1.80)	

^a^ from exact test statistic; ^b^ from t-test of independent samples; *p < 0.01.

**Table 3:** Activities carried out by staff PIP teams (Intervention centers) and wellness champions (Control centers) during the study period, 2008–2012.

Center	Work Organization	Psychosocial Stressors	Musculoskeletal and Ergonomics	Food Environment	Health Improvement
I-1	Communications log	Redesigned employee break room, picnic table	Ergonomics training	Healthy food in vending machine	Yoga, massage
I-2	Employee suggestion box, method to resolve communications problems on units	Picnic tables and lawn furniture	Ergonomics training	Healthy food in vending machine	Nutrition education, walking program
I-3	Staff garden, meetings with certified nursing assistants to discuss health and safety concerns	Staff garden maintenance (for 3 years)	Ergonomics training	Healthy snacks, fruit baskets at each unit, low-cost healthy food options in dining hall	Yoga, weight loss program, nutrition education
C-1	--	--	--	Healthy snacks	--
C-2	--	Softball team	--	Healthy snacks	Annual health fair
C-3	--	--	--	Healthy snacks	--

**Table 4:** Comparison of post-intervention survey responses between PIP and NPHP centers regarding changes in the work environment since the program began.

Work Environment Changes	3 PIP centers: Prevalence (%) (*n* = 331)	3 NPHP centers: Prevalence (%) (*n* = 318)	*p*-Value ^a^
Improved communication between staff and supervisors/management	17% (57)	13% (41)	0.124
Improved communication between co-workers	17% (58)	15% (48)	0.403
More opportunities to participate in decision making	13% (42)	7% (22)	0.014 *
More opportunities to share my opinion (e.g., suggestion box)	13% (43)	9% (28)	0.088
Increased respect	10% (32)	7% (24)	0.336

^a^ from chi-square statistic; ^*^ *p* < 0.05.
